# Discriminating Different Cancer Cells Using a Zebrafish *in Vivo* Assay

**DOI:** 10.3390/cancers3044102

**Published:** 2011-10-31

**Authors:** Karni S. Moshal, Karine F. Ferri-Lagneau, Jamil Haider, Pooja Pardhanani, TinChung Leung

**Affiliations:** Biomedical/Biotechnology Research Institute, North Carolina Central University, North Carolina Research Campus, Nutrition Research Center, 500 Laureate Way, Kannapolis, NC 28081, USA; E-Mails: kmoshal@nccu.edu (K.S.M.); kferrila@nccu.edu (K.F.F.-L.); jhaider@nccu.edu (J.H.); poojapardhanani@yahoo.com (P.P.)

**Keywords:** tumor xenograft, angiogenesis, H1299, CL13, lung carcinoma, cancer cells, zebrafish, subintestinal vessel plexus, PTK787, anti-angiogenesis drug

## Abstract

Despite the expanded understanding of tumor angiogenesis phenomenon and how it impacts cancer treatment outcomes, we have yet to develop a robust assay that can quickly, easily, and quantitatively measure tumor-induced angiogenesis. Since the zebrafish/tumor xenograft represents an emerging tool in this regard, the present study strives to capitalize on the ease, effectiveness, and the adaptability of this model to quantify tumor angiogenesis. In order to test a range of responses, we chose two different tumorigenic cell lines, the human non-small cell lung carcinoma (H1299) and the mouse lung adenocarcinoma (CL13). Non-tumorigenic 3T3-L1 cells served as negative control. The cells were grafted near to the perivitelline space of the zebrafish embryos and the angiogenic response was analyzed using whole-mount alkaline phosphatase (AP) vessel staining and fluorescence microscopy. Angiogenic activity was scored based on the length and number of the newly formed ectopic vessels and the percentage of embryos with ectopic vessels. At 2 day-post-implantation, we detected a significant increase in the length and number of ectopic vessels with H1299 cell implantation compared to CL13 cell transplantation, both are higher than 3T3-L1 control. We also observed a significantly higher percentage of embryos with ectopic vessels with H1299 and CL13 transplantation compared to the 3T3-L1 control, but this parameter is not as robust and reliable as measuring the length and number of ectopic vessels. Furthermore, the systemic exposure of zebrafish embryos to an anti-angiogenesis drug (PTK 787, inhibitor of vascular endothelial growth factor receptor tyrosine kinase) inhibited tumor-induced angiogenesis, suggesting that the assay can be used to evaluate anti-angiogenic drugs. This study implicates the feasibility of using zebrafish xenotransplantation to perform quantitative measurement of the angiogenic activity of cancer cells which can be further extended to measure cancer cell metastasis. This assay represents not only the useful test for patient diagnosis, but also has the potential for evaluating anti-cancer drugs treatment.

## 1. Introduction

Tumor angiogenesis, the process wherein cancer cells can trigger new blood vessel formation, has opened a new field of study in cancer research and greatly expanded our understanding of how certain cellular properties can lead to aggressive cancers and metastasis. Better understanding of tumor angiogenesis and finding ways to block it are significant goals of cancer therapy. The ability to grade the angiogenic potential of different tumor cells takes precedence in clinical diagnostics and warrants the development of a robust assay. Tumor angiogenesis can be mimicked, and thus studied, through xenotransplantation of cancer cells into various animal models [1]. This xenotransplantation involves a procedure where cancer cells are transplanted into a space void of blood vessels, yet close enough to developing blood vessels so that angiogenic factors released by the cancer cells can trigger new blood vessel formation.

Xenotransplantation in mammalian models, such as the mammalian corneal angiogenesis assay and the Matrigel plug assay, are complex, time-consuming, and not easily subjected to genetic manipulation; they often require a skilled technician to perform a microsurgery, can take several months to complete, and cannot easily suppress or activate the host genes after implantation. Their complexity and long running-times also make them expensive and low throughput. However, because they are whole-animal models, they allow the study of host-tumor interactions. Easier, faster, and higher throughput assays are available, such as the chicken chorioallantoic membrane assay (CAM) or yolk sac assay that are considered as *in vivo* or *in vitro* assays when the chicken embryos are grown in Petri dish (*in vitro* assay) for allowing the quantification of blood vessels over a wider area of the CAM membrane than is possible *in ovo*. In addition, genetic manipulation or chemical inhibition in chick embryo is not easily amenable to allow the study of host-tumor interactions. There are several common artifacts that make validation difficult. For example, the CAM membrane is extremely sensitive to increase in oxygen tension and any foreign materials generated during the opening in the shell such as any shell dust particles or shell membrane fragment will cause an inflammation-mediated angiogenic response. For these reasons, the zebrafish/tumor xenotransplantation has been characterized as an alternative model to study tumor angiogenesis [2,3]. This zebrafish model combines the advantages of both types of assays: it is robust, fast, and technically simple, allows for easy genetic manipulation, and is a whole-animal model so that it can be used to study host-tumor environments. In fact, the zebrafish tumor model has been utilized recently to study the genetic pathways of tumor angiogenesis [4-9]. Since the zebrafish embryo's immune system remains immature until 5–7 days-post-fertilization (dpf), and so will not reject the graft during this time, this allows the model to mimic the initial stages of tumor angiogenesis without the use of chemical immunosuppressants [10]. Other advantages include the small size and optical transparency of the zebrafish embryos that allows the disease state to be studied at the whole organism level in real time upon introduction of green fluorescent protein (GFP) expression within the vasculature using the *Tg(flk1:GFP)* transgenic zebrafish strain. More importantly, striking similarities in the molecular and histopathological features of zebrafish and human tumors make it easier to extrapolate the research findings to humans. Another advantage is the permeability of zebrafish embryos to small molecules, thus allowing successful screening of anti-tumor or anti-angiogenic pharmaceuticals. On the other hand, the disadvantage of this zebrafish assay is that it cannot easily be used to study late-stage host-tumor interactions because the developing immune system will start to reject the cells, but this drawback could be overcome by using immunosuppressants.

Despite its many advantages and relatively few disadvantages, the assay lacks sufficient quantification of the angiogenesis observed in response to the zebrafish/tumor xenograft. Until now, this assay qualitatively compares the angiogenic growth and requires side-by-side evaluations of acquired images, or relatively less robust quantitative measurement of angiogenesis. Relying on qualitative methods complicates the comparison of results acquired on different days, even within the same laboratory; and thus makes it nearly impossible to compare results acquired in different laboratories. The development of a quantitative assay will allow standardization by the selection of suitable controls, whose responses could be used to normalize and compare responses observed from test samples, allowing for the comparison of values between assays conducted on different days, by different analysts, and in different laboratories. Standardization will allow cell lines, genetic manipulations, and pharmaceuticals to be evaluated and ranked according to the response observed, and will contribute to maximize the collective scientific effort by eliminating unnecessary duplication of experimental protocols.

Before the assay can be standardized, quantification methods must be established. Since the zebrafish xenotransplantation assay has many potential advantages but still lacks a standard protocol to quantify the result, we chose to make modifications to this assay. Our modifications were employed to maximize its sensitivity range and to develop and evaluate methods for quantifying the angiogenic response. We modeled our studies on the zebrafish/xenograft assay as reported by Nicoli and colleagues. They implanted cancer cells into zebrafish embryos at 2 dpf, and reported angiogenic responses induced by the cancer cells 24 hours later [11]. The tumor-induced angiogenic response was analyzed by sectioning of the stained xenograft to reveal the new vasculature sprouting from the developing subintestinal vessels (SIV) plexus [12] and correlated to the xenograft's angiogenic growth factor overexpression in the transformed cell lines [11]. Here we demonstrated the potential of zebrafish/tumor xenograft model to discriminate angiogenic activity of different cancer cells without introducing the artificial manipulation of growth factor expression. Using 1 dpf embryos for cell transplantation allows 48-hour incubation time before analyzing the angiogenic response and performing robust quantitative measurements.

## 2. Results and Discussion

In the present study, we evaluated three different zebrafish/tumor xenografts by implanting either one of two tumorigenic cell lines, or a control cell line, and then quantifying the subsequent tumor-induced angiogenic response. We chose H1299, which is derived from the human non-small cell lung carcinoma (NSCLC), the most common form of lung cancer, and CL13, a mouse carcinoma cell line derived from A/J mouse lung tumors. The non-tumorigenic cell line 3T3-L1 was used as negative control. Both of these cancer cell lines have endogenous expression of oncogenes, angiogenic factors and cyclo-oxygenase (COX), which are involved in the growth response [13-16]. In particular, they express proto-oncogenes, *TP53* and *Ki-ras*, which regulate tumor growth.

The H1299 cell line is expected to induce a strong angiogenic response because this human cell line is derived from the lymph node metastatic site of a lung carcinoma and several properties can be used to predict its highly proliferative ability and its strong angiogenic response. It secretes the angiogenic peptide hormone, neuromedin B [17,18]; and its angiogenic response involves high expression level of COX proteins [19-21]. The CL13 mouse cell line is also tumorigenic and can induce angiogenic response, thus allowing us to quantify the assay's response in relation to cell lines with different angiogenic potentials [22,23]. It expresses *cox2* weakly as compared to other cell lines derived from lung tumors [22].

We performed the xenotransplantations in zebrafish embryos at 1 dpf specifically to facilitate the detection of angiogenic response of even a weaker tumor cell line in the detectable range. We expected that implanting cells one day earlier would allow a more robust angiogenic response, because it would allow more time to elapse before quantification of angiogenesis. At this stage, the zebrafish embryos have fully developed inter-somitic vessels (ISV), which are formed by angiogenesis, and functional vascular system with the complete circulation of blood cells from the beating heart (ventricle) to the dorsal aorta, through the ISV to the dorsal lateral anastomotic vessel and back through different ISV to the cardinal vein and back to the atrium of the heart [24].

We transplanted CM-DiI dye labeled H1299, CL13, and 3T3-L1 cells, as described [11], at the superficial location of the yolk near to the perivitelline space of the embryos at 1 dpf. One hour after transplantation the embryos were sorted; embryos whose CM-DiI fluorescence pattern indicated that cells had escaped from the primary site of transplantation were discarded. To attain the optimal visualization, we used the *Tg(flk1:GFP)* transgenic zebrafish line, which expresses green fluorescent protein (GFP) under the *flk1/vegfr2* promoter (an early endothelial marker), and exhibits a green fluorescent vasculature [24] in transparent *casper* zebrafish line [25]. We also used whole-mount alkaline phosphatase (AP) vessel staining for easier quantitative measurement without time constraint during documentation using digital imaging and microscopy.

**Figure 1.**
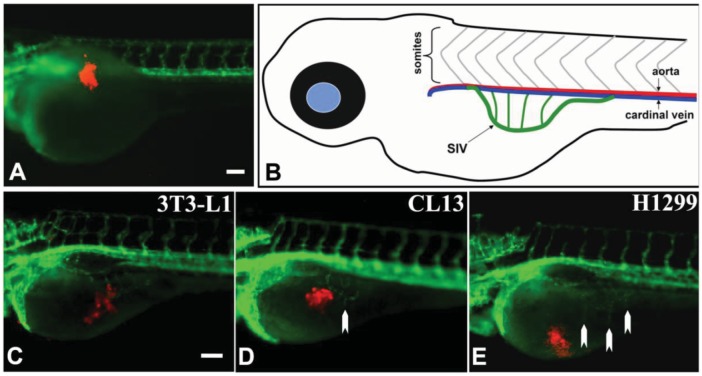
Tumor angiogenesis in transgenic *Tg(flk1:GFP)* zebrafish embryos. Lateral view of a fluorescent transgenic *Tg(flk1:GFP)* zebrafish embryo at 1 dpf that was just transplanted with cancer cells labeled with red fluorescent dye (**A**); (**B**) shows a sketch depicting the SIV (in green) of a 3 day old zebrafish embryo; Lateral views of transgenic *Tg(flk1:GFP)* zebrafish embryos at 3 dpf transplanted with 3T3-L1 cells (negative control) (**C**), CL13 (**D**) and H1299 (**E**) cancer cells; White arrows (**D**, **E**) indicate newly formed ectopic vessels from the SIV. Scale bars = 100 μm.

**Figure 2.**
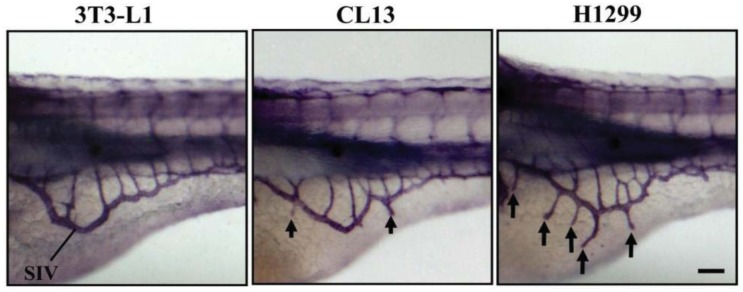
Xenotransplantation-induced neovascularization in the zebrafish embryos. Lateral views of whole-mount alkaline phosphatase stains of zebrafish embryos at 3 dpf were used to evaluate 3T3-L1 control, CL13 and H1299 cancer cell lines for angiogenic potential by measuring newly formed ectopic vessels sprouting from the SIV plexus in the zebrafish/tumor xenograft assay. They show negligible, moderate, and robust angiogenic responses, respectively. Scale bar = 100 μm.

**Figure 3.**
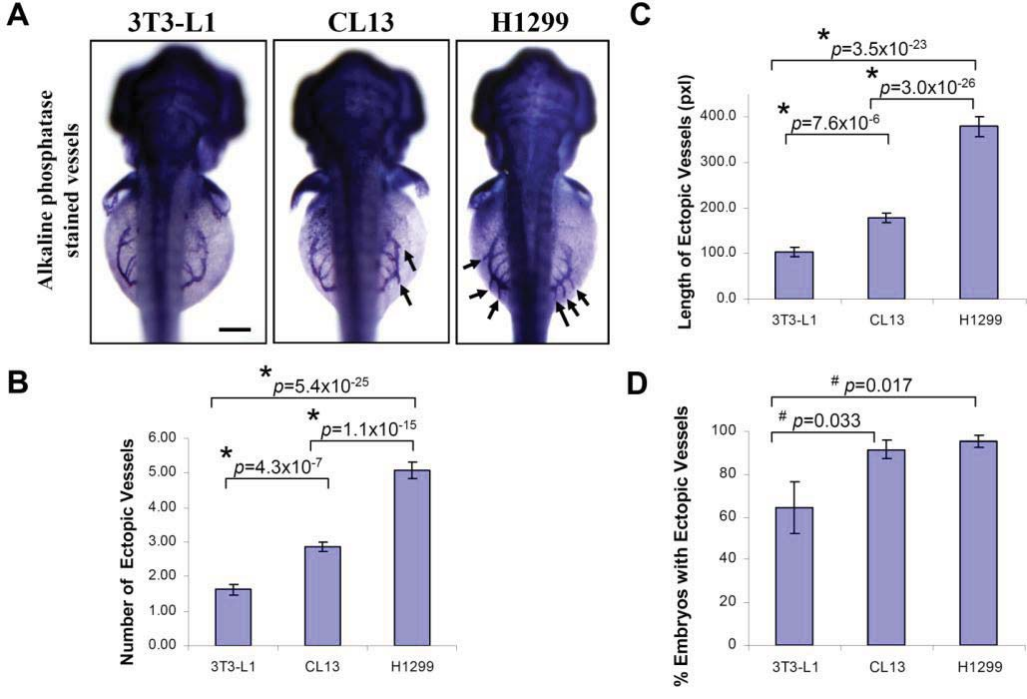
Quantification of angiogenic activity using zebrafish *in vivo* assay. Cells were injected into zebrafish embryos in order to evaluate the ability of zebrafish/tumor xenograft to quantify their angiogenic potentials. (**A**) Shown are representative light microscopy images of zebrafish embryos (dorsal view) at 3 dpf subjected to whole-mount alkaline phosphatase staining, 2 days after implantation with 3T3-L1 control, CL13 and H1299 cancer cells, and used to measure the number (**B**) and the length (**C**) of newly formed ectopic vessels and the percentage of embryos (**D**) with ectopic vessels, showing that H1299 and CL13 cancer cells induced differential neovascular response in the zebrafish embryos as compared to the 3T3-L1 control. The error bars represent ± SEM from three independent experiments. *p* values were determined by the Student's t-test. Both * (*p* < 0.01) and # (*p* < 0.05) indicate statistically significant differences. n represents the number of embryos per group, 3T3-L1 (n = 140); CL13 (n = 198); H1299 (n = 176). Pixel (pxl) corresponds to 0.82 μm. Scale bar = 100 μm.

At 2 day-post-implantation, we observed the newly formed blood vessels sprouting from the SIV plexus using fluorescence microscopy (Figure 1) or alkaline phosphatase staining (Figure 2) followed by imaging, as appropriate, and noted that neovascularization occurred in embryos where cancer cells had been transplanted at 1 dpf [26]. We scored and quantitated the tumor-induced angiogenic response using alkaline phosphatase assay (Figure 3A) and the NIS-Element AR software (Nikon) to calculate the number and length of the ectopic vessels and the percentage of embryos with newly formed ectopic vessels.

We observed a significant increase in the number of ectopic vessels (Figure 3B; H1299: 5.07 ± 0.24; CL13: 2.86 ± 0.15; 3T3-L1: 1.62 ± 0.16) and the length of ectopic vessels (Figure 3C; H1299: 379.1 ± 25.6; CL13: 178.4 ± 11.3; 3T3-L1: 104.3 ± 9.24, unit in pixels) with H1299 cancer cells implantation compared to CL13 implantation. In addition, both of these cancer cells imposed significant difference in length and number of ectopic vessels in comparison to 3T3-L1 control. On the other hand, the percentage of embryos with ectopic vessels is obsolete and not a reliable parameter to distinguish subtle difference between different cancer cells. Although we also observed a significantly greater percentage of embryos with newly formed ectopic vessels in cancer cell transplanted embryos (Figure 3D; H1299: 95.3 ± 2.9%; CL13: 91.6 ± 4.2%) compared to the 3T3-L1 fibroblast injected controls (3T3-L1: 64.2 ± 12.0%). This parameter cannot be used to distinguish between H1299 and CL13 cancer cells.

**Figure 4.**
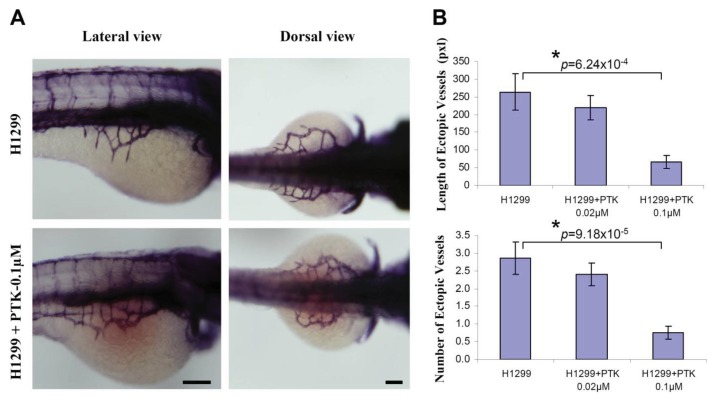
VEGF receptor tyrosine kinase inhibition mitigated tumor angiogenesis in embryonic zebrafish. Cells were stained with CM-DiI before implantation using microinjector. Immediately, after H1299 cancer cell implantation, the embryos were systemically exposed to 0.1 μM PTK 787, pharmacological inhibitor of VEGF receptor tyrosine kinase through 3 dpf. Shown are the representative whole-mount alkaline phosphatase (AP) staining of the zebrafish embryos implanted with H1299 cancer cells, (**A**); Quantification of number and length of the ectopic vessel originating from the developing SIV in H1299 cancer cell implanted embryos with or without PTK787 incubation (**B**). The error bars represent ± SEM. *p* values were determined by the Student's t-test. * (*p* < 0.01) indicate statistically significant difference. n represents the number of embryos per group, H1299 (n = 30); H1299+PTK/0.02 μM (n = 32); H1299+PTK/0.1 μM (n = 29). Pixel (pxl) corresponds to 0.82 μm. Scale bar = 100 μm.

To determine the efficacy of zebrafish xenotransplantation in testing the known protein tyrosine kinase inhibitor (PTK787, a pharmacological inhibitor of VEGF receptor tyrosine kinase) quantitatively on tumor angiogenesis, H1299 cells were fluorescently labeled with CM-DiI and implanted near to the perivitelline space. Immediately, after implantation, the embryos were systemically exposed to 0.1 μM PTK 787 through 3 dpf. By using whole-mount alkaline phosphatase (AP) staining, it was observed that incubation of the H1299 implanted embryos with PTK787 significantly inhibited the H1299-induced number (H1299: 2.87 ± 0.46; H1299+PTK787/0.02 μM: 2.41 ± 0.32; H1299+PTK787/0.1 μM: 0.76 ± 0.18) and the length (Figure 4B; H1299: 264.1 ± 50.9; H1299+PTK787/0.02 μM: 219.8 ± 33.5; H1299+PTK787/0.1 μM: 66.4 ± 17.6) of the ectopic vessels sprouting from the SIV (Figure 4).

In the present study, implantation of human or mouse lung carcinoma cells near to the perivitelline space of the zebrafish embryos induces neovascularization. Using *Tg(flk1:GFP)* transgenic zebrafish, we observed the formation of GFP-labeled blood vessel in response to tumor xenograft in live zebrafish embryos in real time. In addition, the whole-mount alkaline phosphatase (AP) staining detected the newly formed blood vessels after xenotransplantation in wildtype zebrafish embryos. Using this assay, we can compare the angiogenic activity of the cancer cells based on the quantification of tumor-induced neovascular response originating from the developing SIV plexus. This procedure allows 48 hours response time post-xenotransplantation, thereby enhancing the sensitivity to quantitate the angiogenic responses (Figure 1).

We evaluated the angiogenic activity of human (H1299) and mouse (CL13) lung adenocarcinoma cell lines using the zebrafish/tumor xenograft assay. In our experimental conditions, we observed that H1299 cancer cell transplantation induced a robust neovascular response, whereas CL13 cancer cell line is less angiogenic, based on our angiogenesis quantification in the zebrafish embryos. As described by Nicoli and colleagues [11,12], the analyses was primarily based on semi-quantitative evaluation of angiogenic response triggered either by injecting highly angiogenic growth factor (FGF) [12] or the tumor cell variants expressing high levels of FGF [11]. Injecting the angiogenic growth factors or the tumor cell variants is fundamentally different than comparing the angiogenic activity of different naive cancer cells. In the present study, we implanted different naive cancer cells of different origins into high number of zebrafish embryos and compared their angiogenic activity based on the statistical analysis of the length and number of ectopic vessels, which represented the most reliable and consistent parameter for evaluating the angiogenic activity.

Since the large number of embryos can be transplanted and the zebrafish embryos are permeable to the small molecules, the zebrafish bioassay is gaining interest to test and validate various anti-angiogenic molecules. However, there is a pressing need to develop a robust quantitative *in vivo* assay to measure tumor-induced angiogenesis. In this regards, we found that the length and the number of the ectopic vessels are the most reliable and consistent parameters to quantify tumor-induced angiogenesis. In the present study, the embryos were transplanted and systemically exposed to the anti-angiogenesis compound, PTK 787. Based on our *in vivo* quantitative approach, using the length and number of ectopic vessels, we observed a significant inhibition of tumor-angiogenesis. This suggests that zebrafish bioassay of tumor angiogenesis can be used to test and validate various anti-angiogenic compounds based on the robust quantitative measurements of tumor angiogenesis.

## 3. Experimental Section

### 3.1. Chemicals, Reagents, and Cell Lines

Cell culture Dulbecco's Minimal Essential Medium (DMEM), fetal bovine serum (FBS), penicillin/streptomycin, L-glutamine, HBSS, and chloromethylbenzamido-DiI (CM-DiI) were procured from Invitrogen (Carlsbad, CA, USA). The 1-phenyl-2-thiourea, tricaine, trypsin, phosphate buffered saline, Tris, MgCl_2_, NaCl, ethylenediaminetetraacetic acid (EDTA), glycerol, Tween-20 were procured from Sigma-Aldrich (St. Louis, MO, USA). Nitroblue tetrazolium (NBT) and 5-bromo-4-chloro-3-indolyl phosphate (BCIP) were from Roche Applied Science (Indianapolis, IN, USA). Paraformaldehyde (PFA) and methanol (MeOH) was procured from Fisher Scientific (Pittsburgh, PA, USA). PTK787 was procured from Novartis (East Hanover, NJ, USA). The transgenic zebrafish line *Tg*(*flk1:GFP*) expressing green fluorescent protein under the vascular endothelial growth factor receptor 2 (*vegfr2/flk1*) promoter and the transparent *casper* zebrafish was kindly provided from Suk-Won Jin (UNC Chapel Hill, NC, USA) and Leonard Zon (Boston Children's Hospital, MA, USA), respectively. The human non-small cell lung carcinoma (H1299) and mouse lung adenocarcinoma (CL13) cell lines were generously provided by Shengmin Sang (NC A&T State University, NC, USA). The 3T3-L1 cell line was purchased from ATCC (Manassas, VA, USA).

### 3.2. Zebrafish/Tumor Xenograft Model

Zebrafish eggs were incubated at 28.5 °C in 0.3× Danieau's solution (19.3 mM NaCl, 0.23 mM KCl, 0.13 mM MgSO4, 0.2 mM Ca(NO3)_2_, 1.7 mM HEPES, pH 7.0). At 8 hpf, the medium was replaced with 0.3× Danieau's solution containing 1× 1-phenyl-2-thiourea (30 μg/mL) to inhibit the pigmentation. At 24 hpf, zebrafish embryos were screened for green fluorescent vasculature and dechorionated using 150 g/mL of trypsin for 1 hour. The embryos were anesthetized using tricaine (30 to 100 μg/mL) and were transferred onto agar mould for microinjection [26]. H1299 and CL13 cells lines were maintained in Dulbecco's Minimal Essential Medium (DMEM) supplemented with 10% fetal bovine serum and used for microinjection. Before the grafting procedure, cells were labeled with 5 μM of chloromethylbenzamido-DiI (CM-DiI) cell tracker dye. Cancer cells resuspended in serum-free DMEM, were injected at the superficial location of the yolk near to the perivitelline space of the embryos using a FemtoJet microinjector (Eppendorf) with constant injection pressure and injection time. The injection volume and cell suspension was calibrated to be 600–800 cells/injection in each embryo. A micromanipulator MM3301-R, (World Precision Instruments, Sarasota, FL, USA) was used for microinjections using borosilicate glass capillaries (1.0 mm in diameter, World Precision Instruments) which were pulled to inner and outer diameters of 18 and 20 μm, respectively, using a Micropipette Puller (Model P-97 from Sutter Instrument Company, Novato, CA, USA). After transplantation, the embryos were immediately placed at 31 °C. After an hour, embryos with cancer cells injected off the transplantation site were discarded and only embryos with the same amount of fluorescent cancer cells near the future SIV region (95% to 99% of grafted embryos) were kept for analysis. After sorting, the embryos were transferred to 28.5 °C and systemically exposed to pharmacological inhibitor of protein receptor kinase (PTK787), where indicated. All experimental procedures using zebrafish/tumor xenograft research were approved by the Animal Ethical Committee of the North Carolina Central University.

### 3.3. Whole-Mount Alkaline Phosphatase Vessel Staining

At two days post-implantation, the zebrafish embryos were fixed in 4% paraformaldehyde (PFA) and stained for endogenous alkaline phosphatase activity as mentioned elsewhere [11] with modifications. Briefly, the embryos were fixed in 4% PFA for 3 hours at room temperature (23 °C) followed by 5–6 washes in phosphate buffered saline with 0.1% Tween-20 (PBST, pH 7.0). The embryos were dehydrated and made permeable in successive washes of 25, 50, and 75% methanol (MeOH) in PBST, and finally suspended in 100% MeOH. This was followed by serial rehydration of the embryos in 75, 50, and 25% MeOH in PBST and suspended in PBST. The embryos were equilibrated with alkaline phosphatase buffer (100 mM Tris, pH 9.5, 50 mM MgCl_2_, 100 mM NaCl and 0.1% Tween-20) for 30 min at room temperature. Subsequently, the embryos were incubated in staining solution (110 μg/mL NBT and 55 μg/mL BCIP in alkaline phosphatase buffer) at 37 °C until the required staining was attained. The reaction was stopped with 5–6 rinses of stop buffer (PBST with 0.25 mM EDTA, pH 5.5). Embryos were fixed in 4% PFA overnight at 4 °C, then stored in 80% glycerol in stop buffer for further analysis.

### 3.4. Quantitative Analysis of the Tumor Angiogenesis

Zebrafish embryos were imaged using Nikon SMZ1500 fluorescent stereomicroscope with digital color camera DXM1200c (Nikon Instruments, Lewisville, TX, USA). Tumor-induced angiogenesis was quantified using whole-mount alkaline phosphatase staining of zebrafish embryos as described elsewhere [12] with slight modifications. Briefly, the alkaline phosphatase stained embryos were photographed several times at several different angles for the lateral view of the left and right sides of the embryos. The best representative images on each side of the embryos were used and the total number and length of ectopic vessels on both sides were calculated in order to perform quantitation of the ectopic vessel in each embryo. NIS-Element AR software (Nikon Instruments) was used for analysis. The results were from three independent experiments.

### 3.5. Statistical Method

Data presented are mean ± SEM from three independent experiments. The Student's t-test was used to analyze the difference between two groups. *p* < 0.05 was considered as significant. n was total number of embryos per group.

## 4. Conclusions

By using the xenotransplantation procedure on one day old zebrafish embryos, we can discriminate the angiogenic activity of different cancer cells, by the quantitative measurement of the tumor-induced neovascular response originating from the developing SIV plexus. Our study demonstrated the length and number of the ectopic vessels are the most sensitive and consistent parameters for quantification of angiogenic activity of different cancer cells and provide the insight for evaluation of various cancer cells from different grades which could be helpful in characterization of tumors. We demonstrated the feasibility of using zebrafish *in vivo* tumor angiogenesis assay to study cancer-induced vascular remodeling. In a similar scenario, it is expected that zebrafish/tumor xenograft model can also be used as a reliable and sensitive tool to define and quantitate the angiogenic activity of the tumor obtained from various stages during cancer progression and development.
